# Distensibility of Deformable Aortic Replicas Assessed by an Integrated In-Vitro and In-Silico Approach †

**DOI:** 10.3390/bioengineering9030094

**Published:** 2022-02-26

**Authors:** Luigi Di Micco, Giulia Comunale, Stefano Bonvini, Paolo Peruzzo, Francesca Maria Susin

**Affiliations:** 1 Cardiovascular Fluid Dynamics Laboratory HER, Department of Civil, Environmental and Architectural Engineering, University of Padova, 35122 Padova, Italy; luigi.dimicco@dicea.unipd.it (L.D.M.); giulia.comunale@dicea.unipd.it (G.C.); francescamaria.susin@dicea.unipd.it (F.M.S.); 2 Department of Vascular Surgery, Santa Chiara Hospital, 38122 Trento, Italy; stefano.bonvini@apss.tn.it

**Keywords:** aortic distensibility, aortic compliance, aortic phantom, in-vitro experiments, FSI simulation, pulse-duplicator

## Abstract

The correct estimation of the distensibility of deformable aorta replicas is a challenging issue, in particular when its local characterization is necessary. We propose a combined in-vitro and in-silico approach to face this problem. First, we tested an aortic silicone arch in a pulse-duplicator analyzing its dynamics under physiological working conditions. The aortic flow rate and pressure were measured by a flow meter at the inlet and two probes placed along the arch, respectively. Video imaging analysis allowed us to estimate the outer diameter of the aorta in some sections in time. Second, we replicated the in-vitro experiment through a Fluid-Structure Interaction simulation. Observed and computed values of pressures and variations in aorta diameters, during the cardiac cycle, were compared. Results were considered satisfactory enough to suggest that the estimation of local distensibility from in-silico tests is reliable, thus overcoming intrinsic experimental limitations. The aortic distensibility (*AD*) is found to vary significantly along the phantom by ranging from 3.0 × 10^−3^ mmHg^−1^ in the ascending and descending tracts to 4.2 × 10^−3^ mmHg^−1^ in the middle of the aortic arch. Interestingly, the above values underestimate the *AD* obtained in preliminary tests carried out on straight cylindrical samples made with the same material of the present phantom. Hence, the current results suggest that *AD* should be directly evaluated on the replica rather than on the samples of the adopted material. Moreover, tests should be suitably designed to estimate the local rather than only the global distensibility.

## 1. Introduction

In vitro experiments are widely adopted to investigate physiological or pathological hemodynamics, surgical approaches, and the performance of artificial devices [1,2,3,4,5,6]. Most of the time, experimental mock loops host replicas of cardiovascular districts that reproduce both the anatomy and the function of the mimicked organs [7,8,9].

Sometimes replicas are made of rigid materials [10,11,12,13]. However, such an approach is suitable only when the main objective of the experiments is to study the flow patterns in the short regions, i.e., when the effects of pressure waves propagation, due to vessel deformability, can be neglected.

In the most recent literature, deformable replicas produced by 3D printing techniques are increasingly adopted [14], and it is well recognized that they have to mimic the physical characteristics of the real organ, in primis the mechanical behavior, as close as possible [15]. Nevertheless, to our best knowledge, the issue of replicas’ distensibility estimation is far from being appropriately addressed. Some investigations do not assess the compliance of the printed replica [16,17], or just qualitatively verify that the mechanical properties of the adopted material resemble the in-vivo ones [15]. Instead, others evaluate the global response of the phantom. Gallarello et al. [18] tuned the aortic phantom compliance by connecting the prototype to a compliance chamber (Windkessel tank) and adjusting the water level inside the tank. Sparks et al. [19] characterized the overall distensibility of the compliant vascular vessels connected to a flow loop system by using intravascular ultrasound. Finally, in some further cases the compliance (or the distensibility) is not directly estimated on the prototypes but by increasing/decreasing the internal volume of cylindrical tubes made of the same material as the replica (see, e.g., Biglino et al. [20] and Comunale et al. [21]).

All the above examples seem to forget that the assessment of the phantom capability of mimicking the behavior of the real vessel must take into account the strong local interaction between the fluid dynamics and the solid boundaries mechanical response. Moreover, it is worth recalling that the best possible knowledge of the distensibility of the entire prototypes is paramount to suitably design the experiments that focus on the deployment of medical devices, as well as on the analysis of local hemodynamics [22].

Unfortunately, the local definition of the in-vitro distensibility of the vessel needs minimally invasive, continuous, and spatially distributed pressure measurements. This is unfeasible to do, in common practice, without compromising the integrity of the replica. Consequently, we here propose an integrated in-vitro/in-silico system to obtain an exhaustive characterization of the mechanical response of the vessel and focus on the local distensibility of the phantom. For this purpose, we have chosen as a case study the parametrized aortic replica of Comunale et al. [21]. The in-vitro measurements are based on a simple and low-cost imaging analysis technique, and the in-silico data are obtained by the FSI technique proposed in the aforementioned paper.

## 2. Materials and Methods

### 2.1. Experimental Set-Up

**Figure 1:**
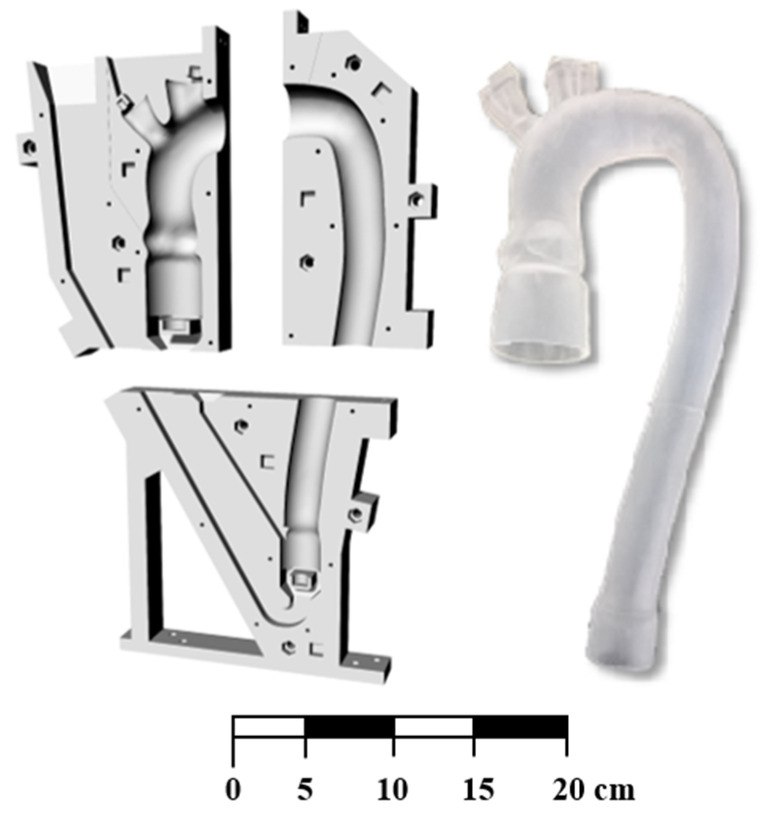
The customized mold to realize the silicone aorta. The mold is divided into six modules.

To create the aortic phantom, the fused deposition modeling method [23] was used to make a customized mold, which was divided into six modules. Inside the external mold, an ABS (Acrylonitrile Butadiene Styrene) core of the inner volume of the vessel was then inserted. The aortic phantom was obtained by simply pouring silicone (Prochima Cristal Rubber, shore-hardness 40) into the mold and then by melting the core with acetone after silicone solidification. The production process enabled the prototyping of a transparent phantom as deformable as the healthy aorta (Figure 1). The obtained parametrized geometry was described by Comunale et al. [21].

The experimental set-up was composed of the parametrized aortic phantom housed in a suitably designed Pulse Duplicator (PD) able to reproduce the systemic circulation. The PD was developed at the Cardiovascular Fluid Dynamics Laboratory of the University of Padova (Italy).

**Figure 2:**
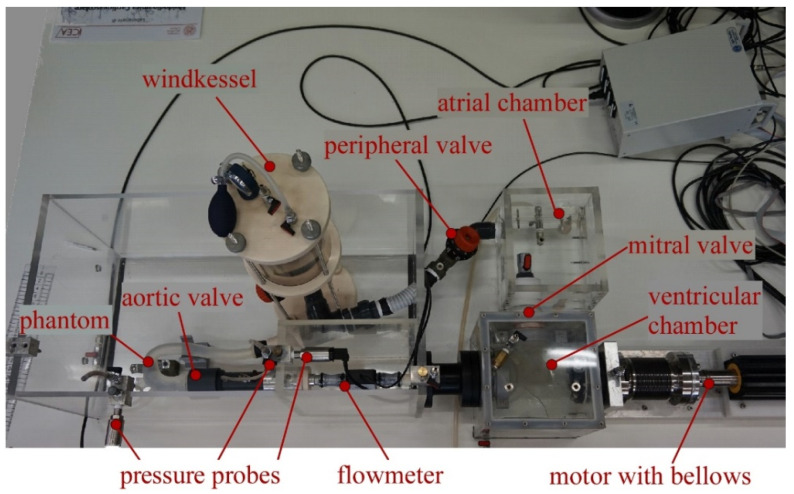
The Pulse Duplicator at the HER Lab. It is composed by the motor with the bellows, the left ventricle, the flowmeter, the aortic valve, the phantom of the aorta, the pressure probes, the systemic resistances, the Windkessel tank, the left atrium, and the mitral valve.

The pulse duplicator harnessed the advantages of the lumped-parameter approach and was successfully used to model the pathophysiological conditions of flows and pressures in cardiovascular circulation [24,25,26]. It was composed of the left ventricle, the aortic valve, the ascending aorta up to the first portion of the descending aorta, the systemic resistances, the left atrium, and the mitral valve (see Figure 2). The left ventricle was mimicked by means of a chamber connected to an electromagnetic motor coupled with a bellows, allowing the reproduction of the ventricular contractility. The ventricular outflow passes through the aortic valve into the phantom. To simulate the compliance of large vessels except for the aorta, a Windkessel tank was placed downstream of the aorta, where a valve was inserted to reproduce the total systemic resistance, i.e., the dissipation through the capillary bed of both the upper and lower body. Finally, the circuit ended in a second chamber mimicking the left atrium, which was connected to the ventricular chamber via the mitral valve. The latter and the aortic valve are two bi-leaflet prosthetic valves namely, Sorin Allcarbon valve (27 mm) and On-X valve (25 mm). Although this configuration did not include the cerebral circulation, its effects on the aorta are minimal, allowing us to study the systemic circulation and, in particular, the aortic hemodynamics [27].

The electromagnetic motor was governed via a PC by means of a LabVIEW program. The software controls the longitudinal position of the bellows that in turn pumps/fills the fluid within the ventricular chamber, thus resulting in a pulsatile flow through the circuit as follows. In systole, the shortening of the bellows promoted the flow through the aortic valve, whereas, in diastole, the bellows recovered their initial length, inducing a flow from the atrial to the ventricular chamber through the atrioventricular valve. It is possible to control the amount of circulating flow and the pressure within the system by varying the shortening amplitude of the bellows, adjusting the air pressure in the windkessel tank, and/or modifying the peripheral valve opening. For more details, see Rampazzo et al. [28].

The working frequency of the bellows varied from 20 to 140 bpm; the overall flow rate results in stroke volume (SV), i.e., the total volume ejected in each heartbeat by the ventricle, between 40 to 90 mL, and in mean aortic pressure between 70 to 150 mmHg, i.e., reproducing physiological ranges for an adult person.

The constructed PD was provided with sensors that measured the continuous-time flow and pressures. In particular, the flow was measured by the ME-PXN Inline Flowsensors 25 mm (Transonic System Inc., Ithaca, NY, USA), placed upstream the aortic valve, whereas the pressures were recorded by three PCB Piezotronics Pressure Transducer—Model 150. The latter was placed at specific locations to capture the pressure upstream and downstream of the aortic valve and at the phantom outlet. For the purpose of this study, two pressure probes are used. One in the brachiocephalic artery (probe p_1_) and the other in the rigid tube downstream of the descending aorta (probe p_2_). Note that the postprocessed pressures and flow rate were obtained by averaging the signals recorded by the sensors over 10 cycles.

### 2.2. The Hydrodynamic Test and Deformation Measurements

We tested the aorta’s prototype in quasi-physiological conditions with saline at 20 °C. To reproduce the desired hemodynamic condition, first, we set the heart rate (HR) equal to 67 bpm, i.e., the heartbeat period is T ≈ 0.9 s. Second, by varying the bellows amplitude and closing the grade of the peripheral valve, we reproduced a total cardiac output (CO) and a mean aortic pressure equal to 4.0 L/min and 100 mmHg, respectively.

A fixed camera (Sony XHR-NX5E, Sony Corporation, Tokyo, Japan) was mounted above the phantom and recorded the aorta movements during the experiment. The image resolution was 1280 × 720 pixels, while the frame rate of the video was 50 fps, i.e., 45 images of the aorta were acquired in a single heartbeat.

The analysis of images from a single camera restricts the quantification of the aorta deformation within the plane of the arch, and, more specifically, it focused on the size of the external diameters of the aortic vessel. The measurement of the lumen size was based on the method proposed by Lanir and Fung [29] for estimating the strain in biaxial tests on specimens of the skin of rabbits. We, hence, marked the phantom with black nail polish, a material deemed appropriate because it does not alter the mechanical characteristics of the silicone, being resistant during the pulsatile load, and opaque to facilitate its detection in the imaging analysis.

**Figure 3:**
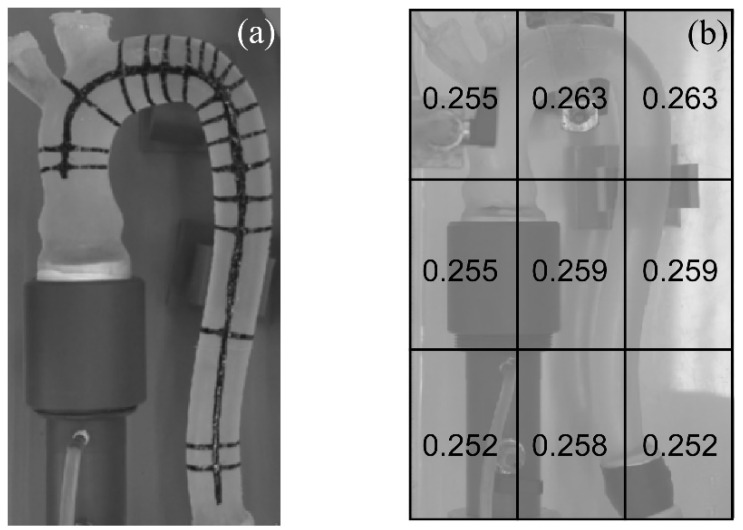
A frame of the silicone aortic phantom tested in the pulse duplicator, (**a**) with fish-bone-shaped configuration marked with black nail polish, and (**b**) with the pixel sizes estimated in six regions of the phantom. The values reported are in mm.

In addition, to suitably reconstruct the overall aorta deformation, particular attention was paid to the definition of the marker’s pattern. The choice of the fish-bone-shaped configuration shown in Figure 3a allowed us to estimate the lateral deformation along the cross-section and to trace translations and shape variations of the aorta.

Inaccuracies in the measurements of the external diameters were ±0.5 mm. The error was due to the pixels’ discretization of the image that has been estimated through a preliminary analysis on a millimeter grid placed in different positions of the area of acquisition (see Figure 3b). The spatial resolution was sufficiently small to correctly describe the prototype contour. Further detailed information on the imaging technique and the measures of the phantom deformations were reported in Appendix A.

### 2.3. The Numerical Analysis

We performed a numerical simulation that reproduced the in-vitro experiment described in Section 2.2. The analysis exploited the Fluid-Structure Interaction (FSI) approach developed in ABAQUS (Abaqus2016—Dassault Systèmes) by coupling the solid model of the aorta with the fluid domain.

We modeled the solid domain with a structured mesh of 15,000 quad shell elements and adopted the implicit solver for the phantom dynamics. The constitutive material of the silicone was represented by the hyperplastic model of Ogden [30], suitably calibrated for a similar analysis in Comunale et al. [21]. The aortic valve was not included in the FSI simulation simplifying the numerical analysis. The wall thickness was uniform and equal to 2.5 mm. To reproduce the real operating conditions, we prescribed null displacements at the tapered connections of the phantom.

**Figure 4:**
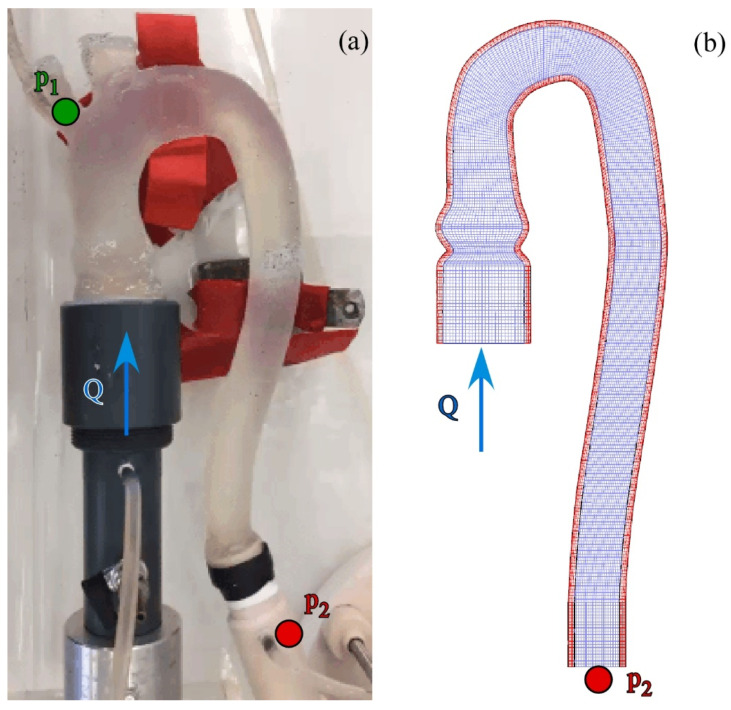
Aorta’s replica of the study. (**a**) Real silicone phantom placed in the Pulse Duplicator, and (**b**) corresponding in-silico models of the aorta (in red and blue the solid and fluid mesh, respectively). The boundary conditions are reported in both panels, i.e., flow rate Q (uniform velocity) at the inlet and time-varying pressure recorded by the probe p_2_ at the outlet. p_1_ reported in (**a**) shows the additional probe placed at the brachiocephalic artery.

The fluid domain was schematized by a structured 3D grid of indicatively 110,000 hexahedral linear elements. The flow through the vessel was governed by the Navier–Stokes equation of a Newtonian incompressible fluid. For this experiment, we assumed the fluid properties of the saline solution at 20 °C, i.e., the flow density and dynamic viscosity was equal to ρ = 1000 kg/m^3^ and µ = 1.01 × 10^−3^ Pa·s. A uniform velocity was prescribed at the inlet as the boundary condition to reproduce the input flow rate measured by the flow sensor. At the outlet of the descending aorta, the in-vitro pressure measured in the PD by the probe p_2_ was set (see Figure 4).

To correctly locate the pressure condition, in the domain of analysis we included a portion of rigid tube of a length equal to about three times the outlet diameter and joined at the end of the phantom. Initially, the fluid was considered at rest and a regular periodic flow into the aorta was achieved in five cardiac cycles. For additional details about the model equations and the calibration coefficients used in the simulation, please refer to Comunale et al. [21].

## 3. Results and Discussion

### 3.1. In-Vitro Measurements

**Figure 5:**
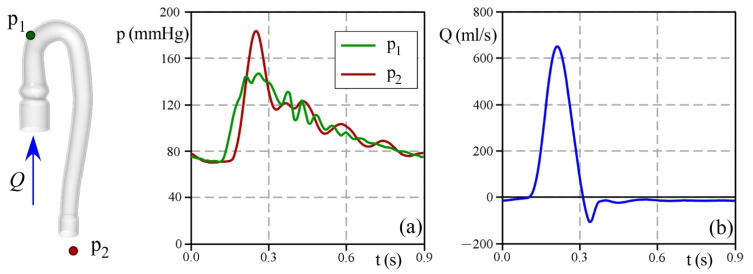
Measures collected in the hydrodynamics test: (**a**) Pressures collected by the probes p_1_ (green line) and p_2_ (red line), and (**b**) flowrate recorded by the flowsensor (blue line).

Figure 5 reports the acquisition of the sensor during the test. In panel a, the probe p_1_ measures a pressure that varies from 70 mmHg at the end of the diastole up to 140 mmHg at the systolic peak, hence consistent with the physiological range. The pressure measured by the outlet probe p_2_ results in both a delay in time and an increase in peak, reaching a maximum value of 180 mmHg. Such a pressure increase is a direct consequence of the Windkessel effect related to aortic compliance.

In panel b, the pulsatile flow has a period of T = 0.9 s with a systolic fraction almost equal to 35% of the total cycle duration. At the systolic peak, the flow rate is about 650 mL/s, while the maximum backflow during valve closing is 100 mL/s. Additionally, a mild leak persists during diastole, likely due to incomplete sealing of the prosthetic valve annulus within the housing in PD. The total stroke volume results in about 70 mL.

**Figure 6:**
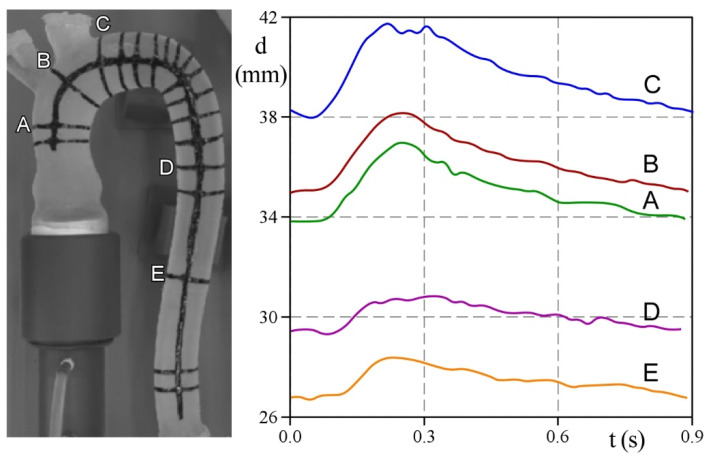
Time-variation of the external diameters during the heartbeat in five sections of interest.

Figure 6 shows the estimates of the external diameter, d, of the aorta during the cardiac cycle for five cross-sections, namely, A, B, and C along the ascending tract, and D and E along the descending tract. A, B, and C display the maximum near the systolic peak time, while changes in lumen size are less emphasized in D and E. It should be noted that deformations in B and C, which are located in the curved region of the aorta, are most likely affected by the effects of curvature on flow patterns. As a consequence, measurements taken in more than one projected diameter would better represent the local wall displacement.

### 3.2. Numerical Outputs

**Figure 7:**
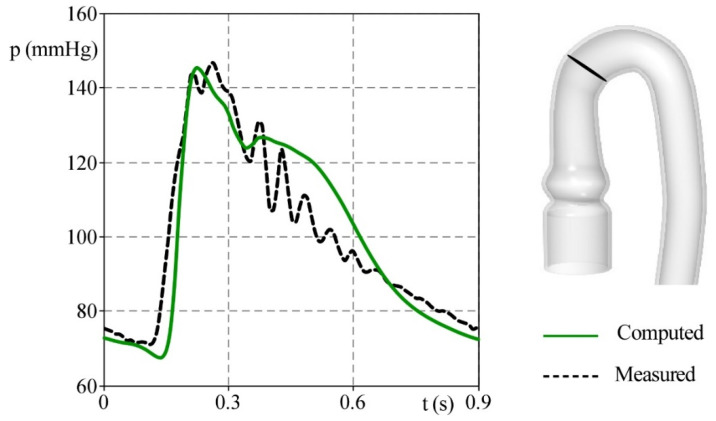
The comparison between the p_1_ pressure measured in the pulse duplicator (black dashed line) and the corresponding values computed by the numerical model (green solid line).

Figure 7 shows the comparison between the pressure values measured by the probe p_1_ and the corresponding ones computed by the numerical model. The two curves match satisfactorily, even if the increase in pressure at the early stage of systole is slightly anticipated in the in-vitro test. During diastole, the two pressures decrease at the same rate, but the numerical phantom damps the overpressures established by closing the aortic valve more effectively.

**Figure 8:**
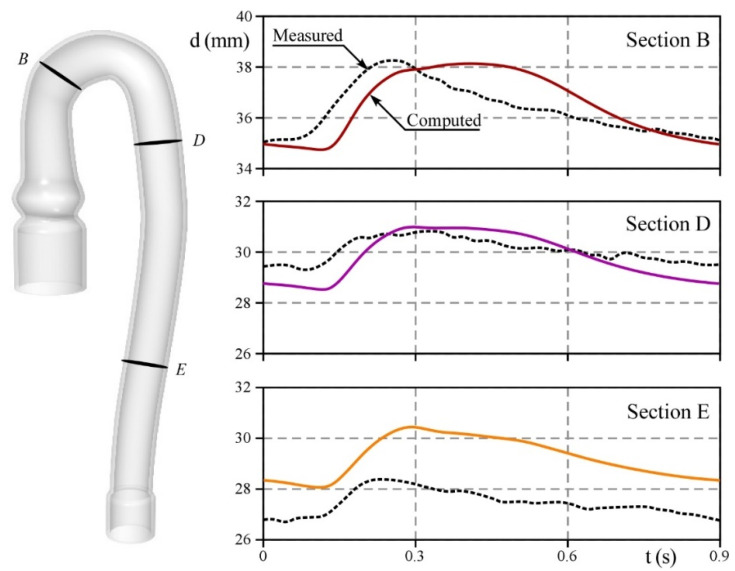
Comparison between the computed (solid lines) and measured (dashed black lines) external diameter in three sections of interest.

To assess the numerical model’s effectiveness in reproducing the deformation of the aortic phantom, during the cardiac cycle, we compare the size of the diameters extracted through imaging analysis, with the projected diameters provided by the numerical simulation and computed in the same plane of the aorta analyzed in the in-vitro test. Figure 8 shows the external diameters calculated in three different sections of the phantom. In the ascending aorta (Section B), for both the in-vitro and in-silico experiments, a minimum and maximum diameter size of 35 and 38 mm is obtained, respectively. Nevertheless, by comparing the in-vitro test with the numerical output, the latter clearly displays a lag in the increase of the lumen size and a longer plateau after reaching the maximum size. At the outlet of the arch (Section D), although a small delay persists in the lumen expansion at the beginning of the ejection, the in-silico and in-vitro diameter size differ in less than 1 mm, ranging from 28.5 to 31 mm and from 29.5 to 31 mm, respectively. Lastly, in the descending aorta (Section E), the computed estimations overrate the measures of the diameter by 2 mm.

Overall, the discrepancies observed between the in-vitro and in-silico tests are related to the timing, shape, and amplitude of the signal. It seems reasonable to suppose that the above is due to the mismatch between the two models in both the geometry and the mechanical response. In particular, the configuration of the phantom lodged in the PD somehow differs from the initial shape adopted in the numerical analysis. As the terminal portion of the prototype is jointed with a rigid tube, the curvature of the descending tract is accentuated. This may result in a non-zero initial stress condition (see Figure 4). With this configuration, the deformation near the phantom outlet is limited, as highlighted in Section E. Moreover, neither we can exclude the presence of spatial defects in both the thickness and the density of the silicone replica, nor we can neglect the effect of induced viscoelasticity owing to the numerical scheme which was set up to ensure the convergence of the model. In conclusion, some differences arise in compliance and inertia of the in-vitro and in-silico aorta. However, the maximum and minimum size of the external diameter, for each section, are satisfactorily predicted by the in-silico model which can hence be adopted to predict them.

### 3.3. Estimation of the Phantom Distensibility

**Figure 9:**
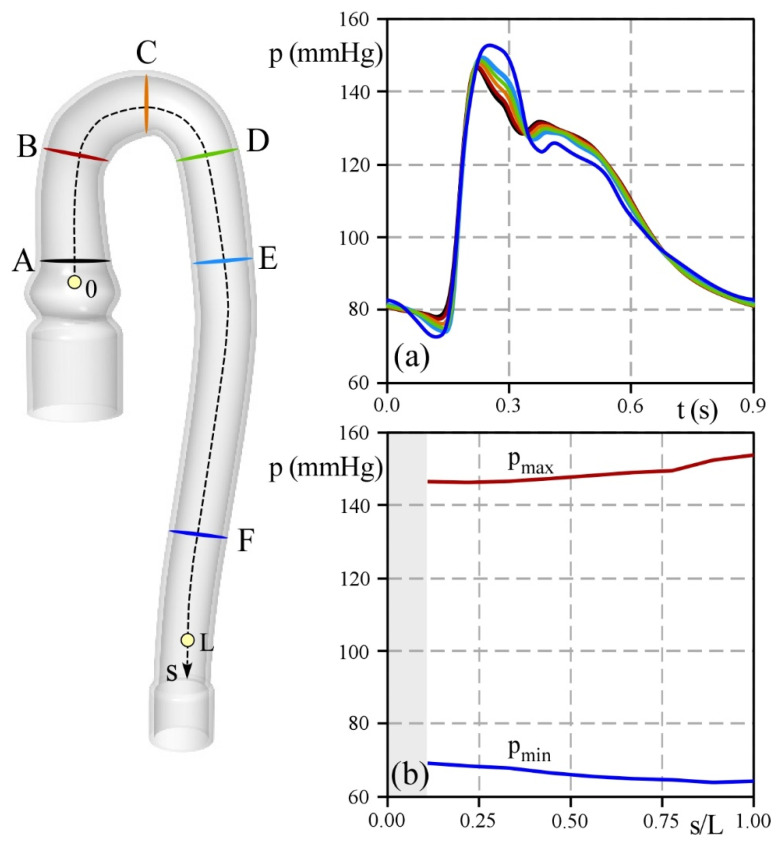
Pressure analysis of the Aorta. (**a**) Time-varying pressure in some sections of the domain, and (**b**) the maximum (red line) and minimum pressures (blue line) calculated along the path line, s/L.

The good agreement between the measured and computed pressure at the probe site in Figure 7 suggests that the numerical model can capture the basic characteristics of the pressure measured in the in vitro test. Accordingly, it can reasonably be assumed that this result is valid for the entire domain. Figure 9 shows the computed pressure waves in six sections along the aortic artery (panel a) and both the maximum, $p_{max}$, and the minimum, $p_{min}$, pressure as a function of the axial position.

In the early phase of systole, pressures grow approximately at a constant rate. However, the maximum value increases moving from the inlet towards the outlet of the aorta, as expected. For instance, in the section closest to the inlet (Section A), the peak is 145 mmHg, whereas near the outlet (Section F) the pressure reaches a maximum of 155 mmHg. Analogously, the minimum pressure recorded in diastole decreases moving downstream from the aortic inlet, displaying a reduction of approximately 8 mmHg. Hence pulse pressure that solicits the wall of the aorta increases along the vessel mainly due to the compliance of the deformable model, as well as the variation in its shape.

**Figure 10:**
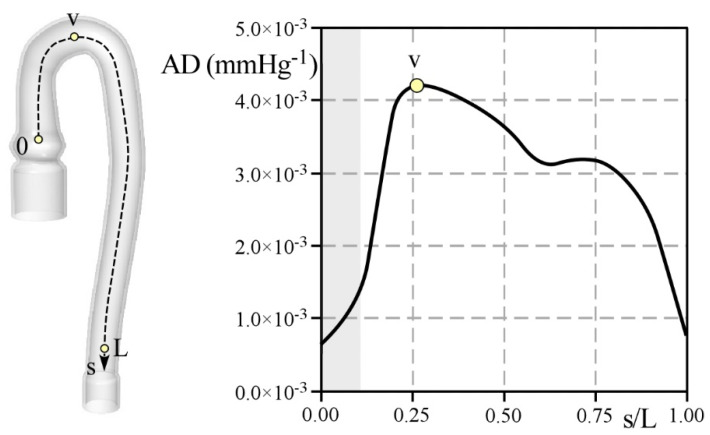
Aortic distensibility, *AD*, estimated along the normalized path line, s/L, according to Equation (1).

Finally, the compliance of the aorta is considered introducing the aortic distensibility, AD (see Figure 10). Locally, AD is estimated in each section as:

(1) $$AD=\frac{2\left(d_{max}-d_{min}\right)}{d_{min}\left(p_{max}-p_{min}\right)},$$

where $d_{max}$ and $d_{min}$ are the maximum and minimum vessel diameter calculated in systole and diastole, respectively, and $p_{max}-p_{min}$ is the pulse pressure during the heartbeat.

Near the tapered connections, i.e., s/L≈0 and s/L≈1, where the phantom is locked to the Pulse Duplicator, the aorta displays an unrealistic, but expected, distensibility AD value close to 0. Moving from the inlet, AD increases reaching its maximum at position v located in the middle of the aortic arch (AD= 4.2 × 10^−3^ mmHg^−1^). Along the descending tract, distensibility decreases and in the last portion of the vessel AD≈ 3.0 × 10^−3^ mmHg^−1^.

Some comments arise from the reported results. First of all, preliminary tests performed by Comunale et al. [21] on a straight cylinder, made with the same material as the present phantom, estimated an AD value of 5.2 × 10^−3^ mmHg^−1^. On the contrary, lower values of AD were obtained in the present analysis, which falls into the physiological range (i.e., from 3.9 × 10^−3^ to 7.5 × 10^−3^ mmHg^−1^ [31,32]) only in a limited portion of the aortic arch. The differences between the expected and actual phantom distensibility clearly show that a reliable estimation of *AD* from preliminary tests on the phantom material is hardly feasible. Rather, the phantom installed in the circuit has to be considered to evaluate the real *AD* and to acknowledge its behavior along the phantom axis.

The assessment of the real behavior of the distensibility is required to successfully investigate problems, such as stent migration and aneurysm development. In fact, the migration dynamics are strictly related to two counterbalanced forces, namely the drag force due to the pulsatile flow and the resistance given by the fixation hooks and friction [33]. The magnitude of these forces depends on the pressure acting on the lumen wall, which depends also on compliance and blood waveform [7,34]. This means that the in-vitro response of a stent varies not only with the hemodynamics working conditions selected (viz., SV, mean pressure, and HR) but also with the position where the stent is implanted, i.e., both the local geometry and distensibility of the replica.

Moreover, it is well established that the onset of aneurismatic lesions along the thoracic aorta is promoted, among other factors, by high and persistent oscillatory wall shear stress [35,36]. However, it is not clear the correlation between the site of the lesion and the local distensibility of the aorta. Hence, in-vitro experiments performed in an aortic replica of known variable distensibility may help in mapping the critical wall shear stress as a function of both the forcing hemodynamic conditions and distensibility.

## 4. Conclusions

The present investigation studies the distensibility, *AD*, of a silicone phantom of the aortic arch under pulsatile flow through an integrated approach that exploits both experimental and numerical analysis under the same operating conditions.

Numerical estimations of both pressure and vessel size were compared favorably with experimental measurements, validating the reliability of the in-silico model to predict the distensibility of the phantom.

The distensibility of the aortic replica ranges from 3.0 × 10^−3^ to 4.2 × 10^−3^ mmHg^−1^ with the maximum located at the middle of the aortic arch. This result not only shows that *AD* is smaller than the value expected from preliminary tests performed on straight cylinders but also exhibits a significant local variability, which is usually neglected in the literature.

It is well known that unsteady fluid flows inside a deformable domain are driven by the interactions that the fluid and the solid boundary continuously exchange at the local scale. Hence, the inclusion of the local distensibility in the case of flows inside cardiovascular replicas guarantees accuracy and precision to the methodological approach.

Accordingly, in-depth knowledge of *AD* can be decisive to correctly analyze the behavior of implanted devices, such as stents, or the formation and evolution of aneurysms. Therefore, appropriate techniques for establishing the distribution of *AD* are required. The integrated approach proposed and discussed here appears simple and reliable for this purpose.

## Figures and Tables

**Figure A1:**
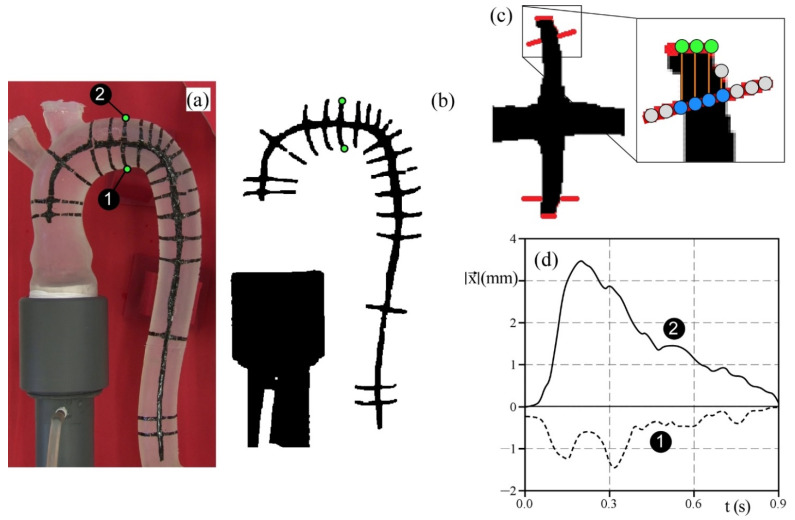
Determination of the edge displacement. (**a**) Acquisition of the phantom image from the camera. (**b**) Segmented image by the morphology function of LabView. (**c**) Detection of the edge marker starting from the manually crossed segment (red lines). Blue circles denote the pixels used by the algorithm to track the edge contour (green circles); gray pixels are pixel selected that do not contribute to the contour definition. (**d**) Example of an estimate of displacements for cardiac cycle for the points 1 and 2 of panel a and b.

## Data Availability

Data are available at http://researchdata.cab.unipd.it/id/eprint/596.

## Appendix A

Figure A1 shows the image process analysis. Preliminarily, the camera is positioned orthogonally to the plane where the phantom is placed, while a red background is set to enhance the recognition of the contour (panel a). Then, the images collected by the camera are post-processed to extract information of the geometry of the aortic arch as follows.

The grey-level morphology function available in LabView creates a binary image by setting a threshold value of the pixels’ digital numbers, to distinguish the objects from the background of the image. The result is a segmented image in which pixels equal to 255 (black color) and 0 (white color) are assigned to the objects and the background, respectively (panel b).

The deformations of the phantom are determined by tracking the displacements of the edge pixels during the cardiac cycle. To obtain consistent information on the variation in shape, the method chosen for analysis is Lagrangian, i.e., the selected pixels are the same for each frame. First, the outer diameters of the aorta are estimated by selecting pairs of specular segments on the axis of the aortic arch [37,38] and considering the pixels contained in each segment (i.e., the red lines in panel c). Second, a LabView algorithm, suitably implemented for this purpose, records as origins the coordinates of the pixels. From each pixel, a line moves from the origin towards the edge of the marking section as long as the algorithm recognizes pixels as black (see green circles in panel c). Finally, the algorithm assigns the coordinates of the edge of the phantom at the last positions that are tracked before encountering a white pixel (see, e.g., points 1 and 2 of Figure A1).

The result is the time-varying positions of the selected edge points and, accordingly, the instantaneous displacements induced by the pulsatile flow (panel d).
